# Telehealth in Home Visiting for New Mothers: Are Outcomes Different if the First Visits Are in Person?

**DOI:** 10.1007/s11121-024-01731-5

**Published:** 2024-10-01

**Authors:** Margaret L. Holland, Dorothy J. Fitch, Drishtant Regmi, Lois S. Sadler

**Affiliations:** 1 Department of Population Health and Leadership, University of New Haven, West Haven, CT USA; 2 Electrical and Computer Engineering and Computer Science Department, University of New Haven, West Haven, CT USA; 3 School of Nursing, Yale University, West Haven, CT USA

**Keywords:** TeleHV, In-person, Home Visiting, Family

## Abstract

Home visiting programs for new parents have a broad range of goals, including improvements in maternal and child health, reductions in child maltreatment, and improvements in child development. Before 2020, few home visits were conducted through phone or video encounters, i.e., telehealth home visiting (teleHV). However, rapid adoption of teleHV was required by the coronavirus disease 2019 (COVID-19) pandemic. To understand how conducting initial visits via teleHV was associated with outcomes, we performed a secondary data analysis to make use of the natural experiment created by COVID-19. Utilizing data from the Nurse-Family Partnership, a US national evidence-based model, we compared outcomes for families whose initial home visits were in person (enrolled 10/2019 to 1/2020; *n* = 7066) to those whose first visits were through teleHV (enrolled 4/2020 to 12/2020; *n* = 14,587). TeleHV at intake was associated with a higher likelihood of elevated depressive symptoms at 12 months (OR = 1.37; 95% CI 1.07, 1.76), a lower likelihood of retention to child’s age 12 months (OR = 0.67; 95% CI 0.58, 0.78), a higher likelihood of early drop from the program (OR = 1.77; 95% CI 1.48, 2.12), and fewer screening assessments completed (*b* =  − 0.06; 95% CI − 0.07, − 0.04). No differences were detected between groups for the likelihood of breastfeeding at child’s age 6 months, elevated intimate partner violence (IPV) risk, 90% of attempted visits completed, or time to attrition. COVID-19 may have led families in different groups to have different experiences during key points of child development; however, as both groups’ involvement in the program occurred primarily during the pandemic, they were both subject to comparable influences. These findings suggest that in-person visits have some advantages in the first months of program involvement.

**Supplementary Information:**

The online version contains supplementary material available at 10.1007/s11121-024-01731-5.

Home visiting programs for new parents provide support, education, and referrals for families facing adversity. In 2021, more than 277,000 US families were served by programs based on 15 home visiting models approved as evidence based in the USA (National Home Visiting Resource Center [NHVRC], 2022). While details vary across programs, family recruitment is often based on socioeconomic risk factors such as low income or young maternal age; recruitment may occur during pregnancy or shortly after childbirth. Families receive regular visits from nurses, social workers, and/or paraprofessionals for periods lasting from 2 to 5 years (NHVRC, 2022). These home visiting programs deliver societal savings of $1.26 to $5.70 per dollar spent (2003 $US; Karoly et al., 2006) and have goals including improvements in maternal and child health, improvements in child development and attachment, and reductions in child maltreatment (NHVRC, 2022).

A key element across home visiting programs is the relationship between the home visitor and the family, which provides the foundation for teaching, mentoring, trust, and collaboration (Roggman et al., 2016). Traditionally, this relationship was established through in-person visits. Visits by telephone or video, known as telehealth home visiting (teleHV), were recommended only as occasional replacements for in-person visits, and only after a relationship between the home visitor and the family was established (Garger, 2019). However, evidence is lacking on the necessity of in-person visits for effective home visiting.

In 2020, the coronavirus disease 2019 (COVID-19) pandemic required rapid adoption of telehealth, including teleHV for home visiting programs. By early April 2020, when shelter-in-place orders were in force in 42 states, 99% of home visits used teleHV (O’Neill et al., 2023). As states reopened, teleHV use continued; in 2021, when over 3 million home visits took place, approximately two-thirds of them occurred through teleHV (NHVRC, 2022). After the federal Public Health Emergency ended in May 2023, the Health Resources and Services Administration clarified that home visiting programs could continue to use federal funds for telehealth visits (Important Home Visiting Information During COVID-19, 2023).

Numerous advantages of teleHV for both families and home visitors have been reported (Hadley et al., 2023). TeleHV can facilitate flexible scheduling and can support visits not tied to a specific location, enabling a productive visit to take place even when a mother is not home (e.g., when she is staying at another home). The ability to connect via teleHV may relieve the pressure felt by clients to prepare their homes before visits (Morrison et al., 2022). Both of these factors may improve visit attendance and retention. Remote visits also prevent the transmission of infection—a concern even in non-pandemic times—which is an advantage for nurse home visitors as well as families.

An advantage for home visitors is the reduction in travel time, which allows them to spend more time on client visits and related activities. However, the advantages of virtual visits must be balanced with the need to build rapport between the home visitor and the family and to accurately assess the physical condition of the child and the safety of the home. Although reports on how teleHV has been used are increasing (Morrison et al., 2022; Roben et al., 2022; Schein et al., 2023), there remains limited evidence regarding how teleHV should be best utilized.

Although some important home visiting outcomes can only be measured years after program completion (Avellar & Paulsell, 2011), some outcomes can be measured within the first 6 to 12 months of the child’s life. For this study, we focused on health outcomes (maternal postpartum depressive symptoms, breastfeeding, and maternal intimate partner violence [IPV] risk) and process outcomes (retention, visit attendance, and screening completion). The health outcomes were chosen because they were objectives of Healthy People 2020 (Office of Disease Prevention & Health Promotion, 2020), they have long-term sequelae (Petruccelli et al., 2019; US Preventive Services Task Force, 2016), and they are associated with economic, racial, and/or ethnic inequities (Basile et al., 2019; Cook et al., 2019; Montgomery et al., 2015; National Center for Health Statistics, 2016). These outcomes also have the potential to change over a relatively short time (up to 12 months after the child’s birth) and to be influenced by the use of teleHV. In addition, these outcomes have previous evidence of program effect (Kitzman et al., 1997; Olds et al., 2004, 2013). The process outcomes of retention, missed visits, and early dropout represent missed opportunities for intervention delivery and are associated with reduced program effectiveness (Holland et al., 2014; Raikes et al., 2006). Lower screening completion is also expected to be associated with reduced program effectiveness, because this results in missed opportunities for direct clinical intervention and referrals to support services.

The Nurse-Family Partnership (NFP), one of 24 evidence-based US home visiting models (Home Visiting Evidence of Effectiveness, 2024), operates both internationally and at 260 sites across the USA. From 2019 to 2023, an average of 1900 low-income first-time mothers enrolled each month in the USA. The participants, who are no more than 28 weeks pregnant at the time of enrollment, receive home visits by trained nurses from pregnancy through the child’s second birthday. The frequency of visits varies: weekly during the first 4 weeks after enrollment and the first 6 weeks after childbirth, monthly during the last 4 months of the program (child’s age of 21–24 months), and every other week for all remaining stages. At intake and subsequent points in time, the NFP National Service Office collects data regarding demographics, visits, screenings (to determine the need for services such as mental health treatment or early intervention for developmental delays), and other outcomes for each enrolled family.

In June 2021, NFP supported the limited resumption of in-person visits after the child was born, based on local COVID-19 conditions and the comfort of both the family and the home visitor (J. Sciandra, personal communication, July 12, 2023). A maximum of 30% of visits were conducted in person during the summer of 2021; this percentage decreased in the fall when COVID-19 cases increased nationwide. In April 2023, the NFP supported the full resumption of in-person visits, recommending against teleHV during pregnancy while approving teleHV visits after birth for families with stable circumstances (Nurse-Family Partnership, 2023). The percentage of NFP home visits that were delivered through teleHV in 2023 decreased from 41.9% in January to 28.6% in June (J. Sciandra, personal communication, July 12, 2023).

The purpose of this study was to examine the importance of early visits being conducted in person by comparing families who enrolled shortly before the pandemic and had their early visits in person to families who enrolled during the pandemic and had their early visits through teleHV. Both groups received the majority of their later visits through teleHV. We compared the two groups on the health and process outcomes described above.

## Methods

We conducted a secondary data analysis utilizing data from NFP sites across the USA to make use of the natural experiment created by COVID-19 shelter-in-place restrictions. We excluded sites with nurses who were reassigned from home visiting work to COVID-19-related priorities (10% of sites, 7% of visits), because those sites were likely to have higher attrition due to this disruption. All other US sites were included (*n* = 235 sites). This dataset included data collected by sites and managed by the NFP National Service Office, which provides guidelines for data collection and oversees data quality.

### Comparison Groups

We created two groups for our analyses:

1. The first group, “in-person intake,” included families who enrolled in the program 3 to 6 months before the transition to teleHV (intake—October 2019 to January 2020, with at least one in-person visit before continuing visits through teleHV; *n* = 7066).
2. The second group, “teleHV intake,” included families who enrolled after the transition to teleHV (intake—April 2020 to December 2020; *n* = 14,587).

Both groups received the majority of their later visits through teleHV but may have received some in-person visits during the summer of 2021.

### Outcomes

The family health outcomes measured included maternal depressive symptoms at child’s age of 12 months, breastfeeding at child’s age 6 months, and maternal IPV risk at child’s age 3 months. Because some families dropped out of the program before they could contribute data for each outcome, sample sizes varied and were lower than those for process outcomes. Maternal depressive symptoms were considered elevated if the participant scored above the threshold for referral on either the Edinburgh Postnatal Depression Scale (EPDS; threshold = 10) or the Patient Health Questionnaire-9 (PHQ-9; threshold = 10). For the EPDS, the threshold of 10 is associated with a sensitivity of 0.85 and a specificity of 0.84 (Levis et al., 2020). For the PHQ-9, the threshold of 10 is associated with a sensitivity of 0.84 and a specificity of 0.81 (Wang et al., 2021). Because two different instruments were used, we dichotomized responses to elevated depressive symptoms or not, based on the thresholds described above. Because these scores were not normally distributed, some combination strategies, such as *z*-score standardization, were not appropriate. Breastfeeding was defined as any breastfeeding at child’s age 6 months, either exclusive or supplemented, based on the question, “Does your child continue to get breast milk?” IPV risk was considered elevated if the participant scored above the threshold for referral based on either the HITS Screening Tool of Domestic Violence plus four additional questions on IPV experience and risk or an alternate screening tool as mandated by individual states. In a systematic review, a sensitivity of 86 to 100% and a specificity of 86 to 99% were reported for the HITS tool (Feder et al., 2009).

Our process outcomes included retention, visit attendance, and screening completion. We note that these process measures are likely related to one another, and therefore, when we interpreted those results, we did not consider them independent findings. Because fewer than 60% of families remain engaged in home visiting programs beyond the child’s first birthday (Duggan et al., 2018), we examined retention to this milestone (a) using a dichotomous indicator of retention and (b) measuring time to attrition. Retention was also examined over the first 90 days, because a pattern of leaving the program very early has been observed (Holland et al., 2018), and these early visits are relevant for our study. For this outcome, we compared families who had 2 to 8 visits and left in the first 90 days to families who had more than 8 visits or stayed longer than 90 days; those with fewer than 2 visits were excluded because of variation in how sites recorded initial enrollment visits. For visit attendance, we examined how often scheduled visits were missed by the client, which was operationalized as the percentage of attempted visits that were not completed from child’s age 6 to 12 months. We included completion of screening questionnaires because, in addition to being related to earlier attrition, (a) screening can lead to important clinical interventions and/or referrals for additional services and (b) lack of screening completion may indicate problems, such as insufficient trust between the home visitor and family or visit time devoted to higher priorities (e.g., helping the family navigate a crisis).

### Covariates

The following variables were used for descriptive statistics and as covariates in multivariable models. We have organized them into the following categories: family structural characteristics, county structural factors, NFP home visitor structural factors, and NFP site structural factors. All covariates were included in multivariable models, except as noted.

Due to their potential impact on the families’ capacity to engage with teleHV, family structural characteristics were included as covariates in all models: maternal age at enrollment, housing (lives in own home, rented or owned, yes/no), maternal primary language (English, yes/no), maternal depressive symptoms at enrollment (EPDS or PHQ-9), and maternal IPV risk (HITS or state-mandated alternative). To account for possible association with attrition, we included maternal race (Black, White, multi-racial, other), maternal ethnicity (Hispanic/Latina, yes/no), paternal involvement (father involved at all, yes/no), marital status (single, living with partner but not married, married), maternal mastery (Pearlin & Schooler, 1967), child sex, and weeks pregnant when prenatal care began. We included maternal student status (enrolled, yes/no) and maternal employment status (working, yes/no) because the COVID-19 pandemic impacted these factors for many families. Because NFP programs provided some families with a $500 gift due to the COVID-19 pandemic, receipt of this gift was included as a covariate due to impact on teleHV access and/or outcomes. For some of these variables, we collapsed additional categories to the definitions described here due to small cells and model convergence issues.

County structural factors were included because of the impact they may have had either on the ability of families to attend visits or their comfort in doing so. The county-level factors accounted for broadband access (American Community Survey Data, n.d.); cellular phone coverage (number of carriers; FCC, 2018); COVID-19 cases per 100,000 (Covid Act Now, 2021); COVID-19 deaths per 100,000; and stay-at-home orders (COVID-19 State and County Policy Orders, n.d.). For COVID-19 cases and deaths, we created 7-day averages to include as covariates: average cases during week of intake, average deaths during week of intake, highest weekly average of cases over first 3 months of child’s life, and highest weekly average of deaths over first 3 months of child’s life.

Because the impact of these county factors might vary based on family characteristics, we examined interaction terms between county-level COVID-19 factors and these maternal characteristics: housing status, primary language, age, race, ethnicity, employment status, and marital status. Interaction terms between broadband access and cellular phone coverage (number of carriers) and the following variables were examined: employment status and marital status. Interactions that did not improve model fit (*p* < 0.20) were sequentially removed for each primary model; of 28 possible interactions, 6 to 15 interactions were retained. Squared terms of continuous variables were added to models as needed to improve model specification for each model; of the 13 continuous variables, 0 to 8 squared terms were added.

Two NFP home visitor structural factors were included as covariates: the home visitor’s years of experience with NFP (home visitor level) and the home visitor turnover (client level). The home visitor’s years of experience with NFP may have been associated with family outcomes due to the individual’s skills and effectiveness in this role, including their confidence in conducting a home visit through teleHV. Home visitor turnover was included because of a previously reported association between turnover and client retention (Holland et al., 2018).

NFP site structural factors were also included to account for possible association with visit attendance. Site structural factors included site size (number of clients served in 2020), length of time providing NFP (years since the agency began providing NFP), and site quality proxy (site-level percent of forms completed of those that are recommended for eligible participants, including the screening tools described above, and additional screening tools from other periods of child development). These variables were available from the NFP administrative records.

### Encounter Methods

Because the methods used to provide teleHV varied over time, we described the encounter methods used for each intake group (Table 3). The encounter method for each visit was initially coded by the home visitor as in-person, phone, video conference, email, text, or other. These options were exclusive; only one modality was recorded. After a review of encounters classified as “other,” we created categories of “drop off” (for supplies, forms, etc.), “written” (cards, notes, door hangers), “telehealth (unspecified),” and “app (unspecified).” As a result of this additional coding, multiple encounter methods were recorded for some visits, e.g., text and drop off, in-person, and phone. In-person visits may have taken place outdoors, as on a porch or sidewalk, observing social distancing recommendations. Video conferences used a variety of apps including Messenger, Duo, Facetime, WhatsApp, WebEx, and Zoom. Email included personal email as well as messages on medical office portals such as Epic.

### Data Analysis

To determine if the families whose first visits took place using teleHV were different from those whose first visits were in person, we conducted bivariate tests for differences in family intake factors between time periods (*t*-tests, χ^2^, non-parametric counterparts as needed). We described teleHV use in each group by summarizing visit frequency, duration, and modality.

For our primary analysis, we used regression to identify associations between teleHV at intake and each outcome. Logistic regression was used for dichotomous outcomes (elevated maternal depressive symptoms, any breastfeeding to 6 months, IPV risk, retention in the program to the child’s first birthday, completion of 90% of attempted visits). Linear regression was used for screenings completed. Time to attrition was examined using survival analysis, with time represented as days since the first visit; the child’s birth was considered as a time-varying covariate because of an increase in attrition shortly after birth (Holland et al., 2014, 2018). For all models, we accounted for clustering by site, as interclass correlations indicated that this was appropriate. We reported both bivariate regression models and multivariable regression models. Multivariable models included all covariates described above; because of our large sample size, it was not necessary to minimize the number of covariates.

We also explored the association between the number of early in-person visits with each outcome by replacing the dichotomous group membership variable with the number of in-person visits during the family’s first 6 weeks in the program (continuous variable with values from 0 to 16 visits).

We examined the dataset for missing values, finding that the most common demographic data missing were maternal school enrollment (5.6%) and employment (5.6%; Supplemental Table 1). Baseline values for two health outcome measures also had missing data (15% for maternal depressive symptoms and 36% for IPV risk). The overall fraction of missing data was 18%. Therefore, we used multiple imputations for all multivariable analyses, with 40 imputations.

### Sensitivity Analyses

We repeated the analyses excluding families who enrolled during April 2020 to determine if the findings were different without this transitional month. Still focusing on the transition time, we repeated the analyses excluding families who left the program within 1.5 months of the start of teleHV (last visit between March 15 and April 30, with a child less than 12 months old). To examine the influence of including families with few early visits, which may signify a lack of engagement with the program, we repeated the analyses excluding families with fewer than four visits during their first 2 months. To isolate the contribution of teleHV only, without the in-person visits that accounted for up to 30% of visits during June and July of 2021, we repeated our analyses limiting inclusion to those families with children born at least 6 months before the resumption of in-person visits, using 6-month measures for outcomes. (For process outcomes based on 6- to 12- month measures, we used 3- to 6- month measures.) We examined cohort effects in the teleHV intake group by using time of enrollment as a continuous predictor instead of the dichotomous teleHV intake variable used for other models.

## Results

Across our two analysis groups, in-person intake (*n* = 7066) and teleHV intake (*n* = 14,587), demographic characteristics were comparable in most respects (Table 1). No differences between groups were both significant and meaningful. The average age at enrollment was just over 23 years. The racial makeup of each group was comparable, as was Hispanic identification, marital status, primary language spoken, housing arrangements, and paternal involvement. The average start time for prenatal care was between 8 and 9 weeks for participants in each group.

**Table 1:** Description of sample, by intake group

Family factors	In-person intake (*n* = 7066)^b^	TeleHV intake (*n* = 14,587)	*p*-value	Effect size (*R*^2^)^c^
	*N* (%) or mean (SD) or median (IQR)	*N* (%) or mean (SD) or median (IQR)		
Age in years (at enrollment)	23.2 (5.4)	23.4 (5.4)	.001	.0005
Race			.06	.0006
White	3391 (52.5%)	6779 (51.0%)		
Black/African American	2311 (35.8%)	4797 (36.1%)		
Multi-racial	441 (6.8%)	1029 (7.7%)		
Other (see sub-categories below)	318 (4.9%)	692 (5.2%)		
American Indian/Alaska Native	150 (2.3%)	349 (2.6%)		
Asian	139 (2.2%)	279 (2.1%)		
Native Hawaiian/other Pacific Islander	29 (0.4%)	64 (0.5%)		
Ethnicity			.22	.0001
Not Hispanic	4584 (67.2%)	9537 (68.0%)		
Hispanic	2242 (32.8%)	4488 (32.0%)		
Enrolled in school	1816 (27.3%)	3469 (25.2%)	.001	.0004
Employed	3171 (47.6%)	5616 (40.7%)	< .001	.0029
Child sex			.63	.00004
Male	2495 (48.1%)	5096 (47.7%)		
Female	2696 (51.9%)	5596 (52.3%)		
Housing			< .001	.0009
Lives in public housing	122 (1.9%)	169 (1.2%)		
Lives with parent or family member	2332 (35.7%)	4506 (33.1%)		
Rents or shares own home or apartment	3117 (47.7%)	7001 (49.9%)		
Owns or shares own home, condominium, or apartment	627 (9.6%)	1342 (9.9%)		
Other	337 (5.2%)	579 (4.3%)		
Father involved			.17	.0001
Daily	4978 (74.8%)	10,459 (75.9%)		
At least once a week (not daily)	558 (8.4%)	1159 (8.4%)		
Less than once a week	370 (5.6%)	684 (5.0%)		
Not at all	753 (11.3%)	1485 (10.8%)		
Primary language			.007	.0005
English	6017 (86.5%)	12,562 (88.0%)		
Spanish	757 (10.9%)	1406 (9.8%)		
Others	181 (2.6%)	309 (2.2%)		
Mastery^a^	15.4 (3.3)	15.4 (3.1)	.25	.0002
Marital status			.021	.0002
Single (never married)	4390 (65.9%)	8976 (65.2%)		
Not married (living with partner)	1054 (15.8%)	2357 (17.1%)		
Married (legal or common law)	1015 (15.2%)	2100 (15.2%)		
Divorced/widowed/separated	200 (3.0%)	340 (2.5%)		
Weeks when prenatal care began	8.5 (4.4)	8.6 (4.2)	.33	.00006
Received $500 gift from NFP	649 (9.2%)	1459 (10.0%)	.057	.0003
Maternal depressive symptoms at enrollment	965 (15.9%)	1627 (13.2%)	< .001	.0012
Maternal IPV risk at enrollment	683 (14.1%)	1103 (12.2%)	.002	.0001

^a^Pearlin Mastery Scale, which ranges from 7 to 28, with higher scores indicating greater mastery (Pearlin & Schooler, 1967)

^b^Sample size shown for all observations included in analysis. Some variables have missing data, as detailed in Supplemental Table 1

^c^For *R*^2^, a small effect size is 0.04 (Ferguson, 2016)

For the in-person intake group, the average number of visits per week by family was higher during the pre-pandemic (in-person visit) time than during the pandemic (*p* < 0.001). For both groups, the average number of visits per week per family was higher in the early pandemic period than in the later pandemic period (*p* < 0.001); the higher frequency of recommended visits in the early portion of the program may have contributed to this difference (Table 2). Across all time periods, the difference in the average number of visits per family per week between teleHV and in-person intake groups was not significant (*p* < 0.60). Overall, families attended a higher percentage of recommended visits in the early pandemic period as compared with the later period (*p* < 0.001); however, the in-person intake group attended a higher percentage of recommended visits in the early pandemic period (*p* < 0.001), while the teleHV intake group attended a higher percentage of recommended visits in the later pandemic period (*p* < 0.001). The average visit duration was longer in the pre-pandemic and later pandemic periods than in the early pandemic period (*p* < 0.001).

**Table 2:** Description of visits attended and visit duration by time period and group

	Number of visits	Average number of visits per week by family	Average percent of recommended visits attended by family	Average visit duration
				Minutes (SD)
Pre-pandemic: primarily in-person				
In-person intake	45,031	0.66	86%	69.2 (52.9)
TeleHV intake	n/a	n/a	n/a	n/a
Early pandemic: teleHV^a^				
In-person intake	118,773	0.53	82%	41.7 (47.0)
TeleHV intake	238,519	0.60	78%	44.0 (47.8)
Later pandemic: some in-person^a^				
In-person intake	34,486	0.34	68%	46.0 (43.3)
TeleHV intake	114,822	0.36	70%	45.5 (47.9)

^a^“Early pandemic” is from March 15, 2020, to May 31, 2021, and “later pandemic” is from June 1, 2021, to the end of the study. In the later pandemic period, in-person visits were allowed due to the availability of the vaccine, although teleHV continued based on local COVID-19 conditions and individual comfort

Among completed visits, the phone was the most common encounter method for both groups (in-person intake, 45.2%; teleHV intake, 56.3%; Table 3). The next most common methods were in-person among the in-person intake group (31.2%) and video among the teleHV group (24.6%). Among visits attempted but not completed, a similar pattern unfolded; the only difference was that video visits were the second most common method for in-person intake (16.6%) as well as teleHV intake (19.0%). The teleHV intake group had more completed encounters by phone, video, drop off, and written, but fewer completed encounters that were in-person and by text (all *p* < 0.001). The teleHV intake group had more attempted, but not completed, encounters by phone and video, but fewer attempted, but not completed, encounters that were in-person, by text, and drop off (all *p* < 0.001; except text *p* < 0.003).

**Table 3:** Home visit encounter method, by intake group

	In-person intake	TeleHV intake	Group difference
	*n* (%)^a^	*n* (%)^a^	*p*-value
Visits completed			
Phone	80,416 (45.2%)	176,532 (56.3%)	< .001
In-person	55,518 (31.2%)	43,919 (14.0%)	< .001
Video	32,228 (18.1%)	77,130 (24.6%)	< .001
Text	7598 (4.3%)	11,631 (3.7%)	< .001
Drop off	1757 (1.0%)	3797 (1.2%)	< .001
Email	374 (0.21%)	593 (0.19%)	.13
Telehealth (unspecified)	73 (0.0004%)	317 (0.10%)	.11
Written	71 (0.0004%)	99 (0.03%)	< .001
App (unspecified)	15 (0.00008%)	25 (0.008%)	.86
Total	177,951	313,724	
Visits attempted but not completed			
Phone	13,954 (58.8%)	31,263 (64.8%)	< .001
Video	3951 (16.6%)	9151 (19.0%)	< .001
In-person	3335 (14.1%)	3034 (6.3%)	< .001
Text	2352 (9.9%)	4453 (9.2%)	.003
Email	145 (0.61%)	255 (0.53%)	.21
Written	84 (0.35%)	132 (0.27%)	.16
Drop off	28 (0.12%)	42 (0.09%)	< .001
Telehealth (unspecified)	11 (0.05%)	105 (0.22%)	.06
App (unspecified)	10 (0.04%)	14 (0.03%)	.36
Total	23,736	48,257	

^a^Percentages add to more than 100% because the coding of “other” resulted in multi-coding

Bivariate analysis comparing outcome occurrences by the teleHV intake group shows a significant difference only for retention to the child’s first birthday (in-person intake, 55.8%; teleHV intake, 48.8%; Table 4). In multivariable models, teleHV at intake was associated with a higher likelihood of elevated depressive symptoms at 12 months (OR = 1.37; 95% CI 1.07, 1.76). TeleHV at intake was also associated with a lower likelihood of retention to 12 months (dichotomous indicator; OR = 0.67; 95% CI 0.58, 0.78) and a higher likelihood of early program dropout (OR = 1.77; 95% CI 1.48, 2.12). TeleHV at intake was associated with fewer screening assessments completed (*b* =  − 0.06; 95% CI − 0.07, − 0.04). No differences were detected between groups for the likelihood of breastfeeding at child’s age 6 months, elevated IPV risk at child’s age 3 months, 90% of attempted visits completed, or time to attrition.

**Table 4:** Primary analyses to determine if each outcome is associated with teleHV use at intake. Logistic regression was used, except as noted

Outcome	In-person intake	TeleHV intake	Bivariate models	Multivariable models^a^
	(*n* = 7066)^b^	(*n* = 14,587)	OR, b, or HR (95% CI)	OR, b, or HR (95% CI)
	*N* (%) or mean (SD) or median (IQR)	*N* (%) or mean (SD) or median (IQR)		
Elevated depressive symptoms at child’s age 12 months	234 (8.7%)	445 (9.0%)	1.04 (0.87, 1.24)	1.37 (1.07, 1.76)*
Breastfeeding to child’s age 6 months	1005 (34.8%)	1927 (33.0%)	0.93 (0.83, 1.03)	0.93 (0.81, 1.07)
Elevated IPV risk at child’s age 3 months	295 (11.0%)	537 (9.9%)	0.89 (0.77, 1.02)	0.97 (0.76, 1.23)
Completed at least 90% of attempted visits from child’s age 6 to 12 months	2544 (58.2%)	5134 (60.3%)	1.09 (0.99, 1.20)	1.15 (0.99, 1.33)
Screenings completed^c^	0.58 (0.32)	0.57 (0.32)	*b*: − 0.02 (− 0.03, − 0.003)*	*b*: − 0.06 (− 0.07, − 0.04)***
Retention				
Retention to child's age 12 months	3624 (55.8%)	6632 (48.8%)	0.75 (0.67, 0.84)***	0.67 (0.58, 0.78)***
Attrition as time to last visit^d^	19.6 (6.9, 28.1)	16.1 (6.5, 21.8)	HR: 1.01 (0.94, 1.09)	HR: 1.02 (0.92, 1.14)
Early drop from program^e^	693 (11.0%)	1,383 (10.3%)	0.94 (0.85, 1.04)	1.77 (1.48, 2.12)***

^*^p < .05, **p < .01, ***p < .001

^a^Models include covariates described in the text (28 covariates, 6 to 15 interactions, 0 to 8 squared terms created from continuous covariates)

^b^Sample size shown for all observations included in the analysis. Some outcome variables have missing data, as detailed in Supplemental Table 2

^c^Linear regression used to estimate the percentage of screenings completed of those the family was eligible for

^d^Survival analysis used to estimate the number of days from enrollment to the last visit, truncated at child’s age 12 months. Mean and IQR include all visits, without truncation

^e^Early drop is defined as attending at least 2 visits but not more than 8 visits and not attending any visits more than 90 days after enrollment

To investigate the effect of variation in the number of early in-person visits, we substituted the number of in-person visits attended in the first 6 weeks of program engagement for the teleHV intake group as the primary predictor variable. For most outcomes, replacing teleHV intake with the number of in-person visits switched the direction of the association, as expected, because fewer in-person visits are consistent with teleHV at intake. However, there were a few meaningful differences (Supplemental Table 3). Number of in-person visits was not associated with maternal depressive symptoms (OR = 1.03; 95% CI 0.96, 1.11), while teleHV at intake was. Number of in-person visits was associated with completing at least 90% of visits attempted (OR = 1.09; 95% CI 1.06, 1.13), while teleHV at intake was not.

### Sensitivity Analyses

These findings were robust for several sensitivity analyses (Supplemental Table 4). The following exclusion criteria were examined one at a time: families who enrolled in April 2020, families who dropped out early (between March 15 and April 30, 2020), families with fewer than 4 visits in their first 2 months in the program, and families whose first child was less than 6 months old at the time in-person visits resumed in June of 2021.

We examined if the outcomes were associated with calendar time in the teleHV group to determine if there were changes over time within the teleHV-only period. There were no significant associations for health outcomes or percentage of screenings completed (Supplemental Table 5). However, for each additional month later enrollment time, there was a greater likelihood of completion of at least 90% of attempted visits (OR = 1.02; 95% CI 1.01, 1.04), lower likelihood of retention to 12 months (OR = 0.96; 95% CI 0.94, 0.98), shorter time to attrition (HR: 0.90; 95% CI 0.88, 0.92), and lower likelihood of early drop (OR = 0.95; 95% CI 0.93, 0.98). These estimates are equivalent to families enrolled 3 months later having a 7% higher likelihood of completing at least 90% of attempted visits; an 11% lower likelihood of retention to 12 months; 13% lower likelihood of early drop, compared to families enrolled earlier; and 0.5% lower likelihood of early attrition on any given day.

## Discussion

We found that the use of teleHV during the first months of engagement with home visiting was associated with some negative outcomes (higher likelihood of maternal depressive symptoms, greater likelihood of attrition, and fewer screening completed). No association was detected for breastfeeding, IPV, or completion of attempted visits. TeleHV for first visits was not associated with improved outcomes. These findings suggest that offering teleHV only after the family has been engaged in home visiting for several months (per current guidelines) may result in better outcomes than allowing teleHV earlier. Our findings are consistent with NFP guidelines to use teleHV only after the family is established in the program. Two of our retention measure findings are consistent with reports of increased attrition when teleHV was used exclusively (Traube et al., 2020), although our other retention measure showed no significant difference.

Our sensitivity analyses showed little variation in results, which reduces our concern that specific choices for variable definitions resulted in these findings. However, our analysis using time as a predictor did reveal some variation in outcomes. Later enrollment in the teleHV period was associated with lower retention to 12 months, a greater percentage of visits completed, and a lower percentage of people who left the program in the first 3 months. This suggests that there may have been improvements in the enrollment process over time that encouraged engagement at the beginning of the program but that did not extend to improved longer-term retention. Our observation of changes in outcomes over time in the teleHV period underscores the importance of documenting how teleHV strategies developed and were modified during this time period.

The regression effect sizes reported here are not so large as to suggest that early teleHV visits should never be allowed. If, for example, prolonged inclement weather or local epidemics preclude initial in-person visits, teleHV is an acceptable alternative. Not allowing such flexibility could prevent some families from ever engaging with the program because of the requirement that they enroll before the third trimester of pregnancy.

Future research is needed to better understand the role of in-person visits at the beginning of a family’s interaction with a home visiting program. If in-person visits are important to building the home visitor-family relationship, it may be helpful to consider how to improve relationship building during teleHV. Other factors beyond relationship building may also need to be identified and examined.

Although it is unlikely that home visiting will experience an extended worldwide teleHV-only period again, understanding how to improve the teleHV experience will be beneficial for its application to other situations. TeleHV may be helpful in rural areas, where travel distances for home visitors may prohibit weekly or biweekly visits or in housing situations where a home visitor is not welcome by some members of the household. In addition, future research should address the potential advantages of teleHV and which families might benefit most from teleHV (or some hybrid model of in-person and teleHV).

Going forward, it will be important to determine how teleHV can best be utilized to improve care. If home visiting is delivered as a mixture of in-person and teleHV visits, clinical programs may be strengthened from evidence-based guidance for applying and tailoring each modality to maximize benefits and health outcomes for families served.

### Limitations

We studied one home visiting model (NFP), which limits generalizability to other models. However, NFP is a large, well-studied model, widely implemented in the USA and internationally, that shares key features with other models. Changes in program delivery may have continued through 2020 as the pandemic evolved and home visitors and families gained experience with teleHV. We found some evidence of changes in the effectiveness of teleHV over time. Therefore, it is possible that the negative outcomes associated with teleHV could have been related, in part, to less effective teleHV during these early months. In addition, teleHV during the pandemic was inherently a different experience than teleHV will be in the future; families who chose to participate during the pandemic may have had different characteristics than families who will participate in the future. We found few meaningful differences in family factors between the two groups at intake, although there may have been other unmeasured factors that we could not control for. Program and parenting experiences during the pandemic were likely to be different than they would have been at other times; however, even though some enrolled before the pandemic began, families in both groups experienced much of the program under pandemic conditions.

### Conclusion

This study is the first to our knowledge to use the COVID-19 pandemic as a natural experiment to evaluate the importance of first visits in home visiting being in person compared to teleHV. Our findings of worse outcomes with teleHV support the continued guidance that early visits should be conducted in person when possible. Using teleHV may provide flexibility that can help families remain engaged while allowing home visitors to work more efficiently, thus leading to better outcomes, but only when used within appropriate guidelines.

## Supplementary Information

Below is the link to the electronic supplementary material.

Supplementary file1 (DOCX 60 KB)

## Data Availability

To protect participant privacy, data sharing is not allowed by our data use agreement with the third party that provided the program data, National Service Office (NSO) for Nurse-Family Partnership and Child First. Data requests can be made to the NSO: https://www.nursefamilypartnership.org. County-level data (COVID, cellular phone coverage, broadband coverage) are publicly available at the sources cited in the manuscript.
