# Supplementary material for: Telehealth in Home Visiting for New Mothers: Are Outcomes Different if the First Visits Are in Person?
Source: Prev Sci. 2024 Oct 1;25(7):1153–63. doi: 10.1007/s11121-024-01731-5 (PMC11519217; doi:10.1007/s11121-024-01731-5)
Supplement: Supplementary file 1 — Supplementary file1 (DOCX 60 KB) [file 11121_2024_1731_MOESM1_ESM.docx]

**Supplemental tables**

| **Supplemental Table 1**  *Description of sample and missing data, by intake group.* | | |  |  |
| --- | --- | --- | --- | --- |
|  | **Total**  (n=21,653) | **In-person intake** (n=7,066) | | **TeleHV intake**  (n=14,587) |
| Age in years (at enrollment) |  |  | |  |
| Missing | 22 (0.10%) | 10 (0.14%) | | 12 (0.08%) |
| Race |  |  | |  |
| Missing | 1895 (8.8%) | 605 (8.6%) | | 1,290 (8.8%) |
| Ethnicity |  |  | |  |
| Missing | 802 (3.7%) | 240 (3.4%) | | 562 (3.9%) |
| Enrolled in school |  |  | |  |
| Missing | 1207 (5.6%) | 407 (5.8%) | | 800 (5.5%) |
| Employed |  |  | |  |
| Missing | 1207 (5.6%) | 407 (5.8%) | | 800 (5.5%) |
| Child sex |  |  | |  |
| Missing | 5770 (26.6%) | 1,875 (26.5%) | | 3,895 (26.7%) |
| Housing |  |  | |  |
| Missing | 1521 (7.0%) | 531 (7.5%) | | 990 (6.8%) |
| Father Involved |  |  | |  |
| Missing | 1207 (5.6%) | 407 (5.8%) | | 800 (5.5%) |
| Primary Language |  |  | |  |
| Missing | 421 (1.9%) | 111 (1.6%) | | 310 (2.1%) |
| Mastery |  |  | |  |
| Missing | 4914 (22.7%) | 1,461 (20.7%) | | 3,453 (23.7%) |
| Marital Status |  |  | |  |
| Missing | 1221 (5.6%) | 407 (5.8%) | | 814 (5.6%) |
| Weeks when prenatal care began |  |  | |  |
| Missing | 2463 (11.4%) | 800 (11.3%) | | 1663 (11.4%) |
| Received $500 gift from NFP |  |  | |  |
| Missing | 0 (0.0%) | 0 (0.0%) | | 0 (0.0%) |
| Maternal depressive symptoms at enrollment |  |  | |  |
| Missing | 3292 (15.2%) | 1006 (14.2%) | | 2286 (15.7%) |
| Maternal IPV risk at enrollment |  |  | |  |
| Missing | 7812 (36.1%) | 2233 (31.6%) | | 5579 (38.2%) |

| **Supplemental Table 2**  *Missing data in outcome variables, by intake group.* | | |  |  |
| --- | --- | --- | --- | --- |
| **Outcome** | **Total**  (n=21,653) | **In-person intake** (n=7,066) | | **TeleHV intake**  (n=14,587) |
| Elevated depressive symptoms at child's age 12 months |  |  | |  |
| Missing | 3,292 (15.2%) | 1,006 (14.2%) | | 2,286 (15.7%) |
| Breastfeeding to child's age 6 months |  |  | |  |
| Missing | 12,927 (59.7%) | 4,175 (59.1%) | | 8,752 (60.0%) |
| Elevated IPV risk at child's age 3 months |  |  | |  |
| Missing | 13,525 (62.5%) | 4,382 (62.0%) | | 9,143 (62.7%) |
| Completed at least 90% of attempted visits from child's age 6 to 12 months |  |  | |  |
| Missing | 8,771 (40.5%) | 2,697 (38.2%) | | 6,074 (41.6%) |
| Screenings completed^c^ |  |  | |  |
| Missing | 3,088 (14.3%) | 1,090 (15.4%) | | 1,998 (13.7%) |
| Retention |  |  | |  |
| Retention to child's age 12 months |  |  | |  |
| Missing | 1561 (7.2%) | 566 (8.0%) | | 995 (6.8%) |
| Attrition as time to last visit |  |  | |  |
| Missing | 1,593 (7.4%) | 571 (8.1%) | | 1,022 (7.0%) |
| Early drop from program |  |  | |  |
| Missing | 1,969 (9.1%) | 747 (10.6%) | | 1,222 (8.4%) |
|  |  |  | |  |

Note: for outcomes that require families to be engaged in the program to a certain length of time or child age, families who did not remain engaged until that milestone are included in the number of missing values.

**Supplemental Table 3**

*Sensitivity analyses to determine if each outcome is associated with the number of in-person visits over the first 6 weeks, instead of teleHV intake. Logistic regression was used, except as noted.*

| Outcome | OR (95% CI) | b or HR (95% CI) |
| --- | --- | --- |
| Maternal depressive symptoms at child's age 12 months | 1.03  (95% CI: 0.96, 1.11) |  |
| Breastfeeding to child's age 6 months | 1.00  (95% CI: 0.97, 1.04) |  |
| Elevated IPV risk at child's age 3 month | 1.04  (95% CI: 0.99, 1.1) |  |
| Completed at least 90% of attempted visits from child's age 6 to 12 months | 1.09  (95% CI: 1.06, 1.13)*** |  |
| Screenings completed^c^ |  | b = 0.05  (95% CI: 0.05, 0.06)*** |
| Retention |  |  |
| Retention to child's age 12 months | 1.36  (95% CI: 1.31, 1.4)*** |  |
| Attrition^a^ |  | HR: 0.90 (0.88, 0.92)*** |
| Early drop from program^b^ | 0.52  (95% CI: 0.49, 0.55)*** |  |

*p<.05, **p<.01, ***p<.001

^a^ Survival analysis used to estimate the number of days from enrollment to last visit.

^b^ Early drop is defined as attending at least 2 visits but not more than 8 visits and not attending any visits more than 90 days after enrollment.

^c^ Linear regression used to estimate the percentage of screenings completed of those the family was eligible for.

**Supplemental Table 4**

*Multivariable regressions examining the association between teleHV at intake and the indicated outcomes for each of the sensitivity analyses.*

|  | **Excluding families who:**  **Enrolled in April 2020** | **Excluding families who:**  **Dropped out between 3/15/20 and 4/30/20** | **Excluding families who:**  **Had child < 6 m by resumption of in-person visits (6/1/21)** | **Excluding families who:**  **Completed<4 visits in first 2 months** |
| --- | --- | --- | --- | --- |
| **Outcome** | OR or b (95% CI) | OR or b (95% CI) | OR or b (95% CI) | OR or b (95% CI) |
| Maternal depressive symptoms at child's age 12 months | 1.32 (1.02, 1.71)* | 1.37 (1.07, 1.76)* | 1.37 (1.08, 1.74)* | 1.39 (1.06, 1.82)* |
| Breastfeeding to child's age 6 months | 0.94 (0.81, 1.09) | 0.93 (0.81, 1.07) | 1.01 (0.85, 1.2) | 0.98 (0.84, 1.14) |
| Elevated IPV risk at child's age 3 months | 0.98 (0.76, 1.26) | 0.97 (0.76, 1.24) | 1.04 (0.75, 1.44) | 0.91 (0.69, 1.19) |
| Completed at least 90% of attempted visits from child's age 6 to 12 months | 1.17 (0.995, 1.37) | 1.15 (0.99, 1.33) | 1.01 (0.87, 1.18) | 1.1 (0.95, 1.27) |
| Early drop from program^b^ | 1.87 (1.55, 2.26)*** | 1.77 (1.48, 2.13)*** | 2.13 (1.67, 2.71)*** | 1.49 (1.10, 2.02)* |
| Retention |  |  |  |  |
| Retention to child's age 12 months | 0.65 (0.56, 0.77)*** | 0.63 (0.54, 0.72)*** | 0.66 (0.57, 0.77)*** | 0.68 (0.57, 0.80)*** |
| Attrition^a^ | HR: 0.97 (0.86, 1.08) | HR: 1.01 (0.91, 1.13) | HR: 0.98 (0.88, 1.10) | HR: 1.05 (0.94, 1.18) |
| Screenings completed^c^ | b = -0.05  (-0.07, -0.04)*** | b = -0.06  (-0.08, -0.05)*** | b = -0.09  (-0.11, -0.08)*** | b = -0.05  (-0.06, -0.03)*** |

*p<.05, **p<.01, ***p<.001

^a^ Survival analysis used to estimate the number of days from enrollment to last visit.

^b^ Early drop is defined as attending at least 2 visits but not more than 8 visits and not attending any visits more than 90 days after enrollment.

^c^ Linear regression used to estimate the percentage of screenings completed of those the family was eligible for.

**Supplemental Table 5**

*Calendar time (in months) as the key independent variable predicting each of the following outcomes.*

| **Outcome** | | **OR (95% CI)** | **b or HR (95% CI)** |
| --- | --- | --- | --- |
| Maternal depressive symptoms at child's age 12 months | | 0.96 (0.92, 1.004) |  |
| Breastfeeding to child's age 6 months | | 0.98 (0.96, 1.006) |  |
| Elevated IPV risk at child's age 3 month | | 0.99 (0.94, 1.03) |  |
| Completed at least 90% of attempted visits from child's age 6 to 12 months | | 1.02 (1.01, 1.04)* |  |
| Screenings completed^c^ | |  | b: 0.002 (-0.001, 0.004) |
| Retention | |  |  |
| Retention to child's age 12 months | 0.96 (0.94, 0.98)*** |  |  |
| Attrition^a^ |  | HR: 1.00 (0.98, 1.02) |  |
| Early drop from program^b^ | 0.95 (0.93, 0.98)** |  |  |
|  | |  |  |
|  | |  |  |

*p<.05, **p<.01, ***p<.001

^a^ Survival analysis used to estimate the number of days from enrollment to last visit.

^b^ Early drop is defined as attending at least 2 visits but not more than 8 visits and not attending any visits more than 90 days after enrollment.

^c^ Linear regression used to estimate the percentage of screenings completed of those the family was eligible for.
